# Identification of Use Cases, Target Groups, and Motivations Around Adopting Smart Speakers for Health Care and Social Care Settings: Scoping Review

**DOI:** 10.2196/55673

**Published:** 2025-01-13

**Authors:** Sebastian Merkel, Sabrina Schorr

**Affiliations:** 1 Faculty of Social Science Ruhr University Bochum Bochum Germany

**Keywords:** conversational agents, smart speaker, health care, social care, digitalization, scoping review, mobile phone

## Abstract

**Background:**

Conversational agents (CAs) are finding increasing application in health and social care, not least due to their growing use in the home. Recent developments in artificial intelligence, machine learning, and natural language processing have enabled a variety of new uses for CAs. One type of CA that has received increasing attention recently is smart speakers.

**Objective:**

The aim of our study was to identify the use cases, user groups, and settings of smart speakers in health and social care. We also wanted to identify the key motivations for developers and designers to use this particular type of technology.

**Methods:**

We conducted a scoping review to provide an overview of the literature on smart speakers in health and social care. The literature search was conducted between February 2023 and March 2023 and included 3 databases (PubMed, Scopus, and Sociological Abstracts), supplemented by Google Scholar. Several keywords were used, including technology (eg, voice assistant), product name (eg, Amazon Alexa), and setting (health care or social care). Publications were included if they met the predefined inclusion criteria: (1) published after 2015 and (2) used a smart speaker in a health care or social care setting. Publications were excluded if they met one of the following criteria: (1) did not report on the specific devices used, (2) did not focus specifically on smart speakers, (3) were systematic reviews and other forms of literature-based publications, and (4) were not published in English. Two reviewers collected, reviewed, abstracted, and analyzed the data using qualitative content analysis.

**Results:**

A total of 27 articles were included in the final review. These articles covered a wide range of use cases in different settings, such as private homes, hospitals, long-term care facilities, and outpatient services. The main target group was patients, especially older users, followed by doctors and other medical staff members.

**Conclusions:**

The results show that smart speakers have diverse applications in health and social care, addressing different contexts and audiences. Their affordability and easy-to-use interfaces make them attractive to various stakeholders. It seems likely that, due to technical advances in artificial intelligence and the market power of the companies behind the devices, there will be more use cases for smart speakers in the near future.

## Introduction

### Background

In the context of ongoing public debates on artificial intelligence (AI), dialogue systems or conversational agents (CAs) are receiving increasing attention. Their potential applications are being discussed in various fields, including health care [1,2] and social care [3]. CAs have been used in both fields for several years, but recent developments in AI have fueled the scientific discourse [4,5]. The developments in the field of machine learning and natural language processing (NLP), as well as the success of commercially available CAs, such as Amazon’s Alexa or Apple’s Siri, have been particularly decisive in this regard.

The use of CAs is not limited to a single context; rather, they are used in a variety of settings, including those pertaining to the acquisition of information related to health [6]. CAs using NLP offer a number of features that can be implemented in a variety of health care and social care settings. The field of AI has witnessed considerable progress in recent years, with speech recognition (SR) and NLP advancing significantly. This has enabled the processing of medical terminology in various settings [7]. Although SR in health care has a long tradition dating back to the 1980s, when initial attempts were made to dictate doctor’s letters [8], CAs offer multiple additional features. In the context of hands-free interaction, CAs have been used for the purposes of medication reminders [9], symptom management [10], documentation [11], or communication between patients and nurses or doctors, covering multiple medical fields. These include diabetes care [12], monitoring of pregnant women [13], children with special health care needs [11], hearing tests [14], cardiovascular disease [15], and the support of persons with dementia, to name a few [16].

### The Rise of Smart Speakers

The term “CA” is not clearly defined, and within the literature, multiple synonyms are used interchangeably. These include “virtual assistants,” “AI-driven digital assistants,” “voice-based assistants,” “voice-controlled intelligent personal assistants,” and others. In the study by Laranjo et al [1], the term “CA” is defined as encompassing a range of technologies, including chatbots, embodied CA, which involves a computer-generated character such as an avatar, and smart conversational interfaces, such as Apple’s Siri or Amazon’s Alexa. In order to characterize CAs, the authors propose that it is necessary to differentiate between the type of technology in question (eg, if the software application is delivered through a mobile device or the telephone), the type of dialogue management (finite-state, frame-based, or agent-based), the actors with control over the dialogue initiative (the user, the system, or a combination of both), the input or output modality (spoken or written, or visual in the case of the output), and whether the system is task-oriented or not [1].

This paper is particularly interested in the use of CAs that are embodied in a physical stationary artifact, which is referred to as a smart speaker. Examples of such devices include Amazon’s Echo and Apple’s HomePod. Smart speakers are typically confined to a specific location and serve as a platform for a smart conversational interface or AI-driven digital assistant that can be operated through voice input. In the case of the Echo, this is “Alexa”, while in the HomePod, it is “Siri”. Such assistants are capable of fulfilling a range of tasks, including answering simple questions, switching on lights in conjunction with a smart home system, and playing music. The devices are equipped with one or multiple microphones and software that is capable of analyzing and generating spoken language. In order to operate the devices, the user must utter a designated wake word, such as “Alexa” or “Computer” in the case of Amazon’s Echo [17].

The diffusion of smart speakers has been observed to be high in private households in Europe and North America. Amazon launched the first smart speaker in the United States in 2015. As of 2022, approximately 35% of the total US population had used smart speakers [18]. In comparison to the figures from 2019, this represents an increase of 11.1% [19]. A number of studies conducted by market research companies in other countries have reached similar conclusions. For instance, these studies have found that 33% of internet households in the United States, 34% in the United Kingdom [20], and approximately 12%-33% of all households in Germany own at least one smart speaker [21,22].

A recent study by Gaspar and Neus [23] of smart speaker users in the United States, United Kingdom, and Germany shows that Amazon is still the current market leader (United States: 58%; United Kingdom: 71%; and Germany: 68%) followed by Google (United States: 34%; United Kingdom: 22%; and Germany: 25%) and other brands (United States, United Kingdom, and Germany: 7%). It was also found that in all countries, at least 40% (United States: 46%; United Kingdom: 40%; and Germany: 44%) of respondents use smart speakers several times a day. Participants were also asked about the attractiveness of certain application scenarios, including medical diagnosis. Here, participants gave high ratings: United States (19% very attractive and 36% attractive), United Kingdom (12% very attractive and 34% attractive), and Germany (13% very attractive and 35% attractive).

In light of the commercial success of smart speakers and the aforementioned technological advantages in SR and NLP, there has been a growing body of literature on smart speakers in different health care and social care settings [1,24-27]. Commercial devices, such as Amazon’s Echo, offer a multitude of features. These devices can be used without any direct contact, are relatively inexpensive and easy to operate, and can be customized and personalized by installing new applications and features [28]. These factors have played a pivotal role in the dissemination of the technology. Finally, the widespread adoption of the technology was driven by the pandemic and the subsequent shift in clinical practices toward greater reliance on digital technologies [29]. Nevertheless, the pervasive use of these devices has also given rise to a multitude of issues and concerns, most notably data collection, storage, and protection [8].

Hence, the devices have attracted increasing attention, with several reviews on CAs in health care settings having been published recently. Each of these reviews has a specific focus: these include, for instance, design and evaluation challenges [30], effectiveness and usability [31], or chronic conditions [32,33]. To the best of our knowledge, no review has been conducted to date that specifically examines the use of smart speakers within health care and social care settings.

As evidenced by the current state of research, smart speakers are becoming increasingly prevalent in the field of health care and social care. However, there is currently no systematic review available that specifically investigates use cases, settings in which the devices are used, or target groups. To address this gap, our main research question is as follows: What are the scenarios of the use of smart speakers in health care and social care? To address this research question, the main aim of this paper is to present a review of the current research on the use of smart speakers in health care and social care.

## Methods

### Overview

In order to provide an overview of the existing literature on smart speakers in health care and social care, we conducted a scoping review. The main aim of this approach is to observe, synthesize, and understand current trends [34]. In contrast to a systematic review, which is more suitable for the presentation of a specific clinical question or the presentation of evidence for practice, a scoping review is particularly suitable for identifying features and concepts. Furthermore, it does not aim to provide a synthesizing result for a specific question but rather to provide an overview of a specific topic [34,35]. Thus, the scoping review is a particularly suitable instrument for analyzing the research interest. This encompasses the identification of the nature of the literature, the collation of information on key topics, and the identification of knowledge gaps [35]. Its methodological framework was first published by Arksey and O’Malley [36] and later adapted by Levac, Colquhoun, and O’Brien [37]. Contrary to a systematic review, search terms can be adjusted along the process of a scoping review [36,38]. For the conduction of the present review, the guidelines of Peters et al [39], the Preferred Reporting Items for Systematic Reviews and Meta-Analyses (PRISMA) [40] and its extension for Scoping Reviews (PRISMA-ScR) [41] were followed. The results were presented according to the PRISMA checklist (Multimedia Appendix 1).

### Search Strategy and Selection Criteria

The literature search was conducted between February 2023 and March 2023. This included a systematic literature search of 3 databases (PubMed, Scopus, and Sociological Abstracts) and a cross-search of the first 20 pages of Google Scholar. This was supplemented by tracing reference lists for further relevant studies. We used the program Citavi 6 for literature management. The review protocol is available on request from the authors. The following keywords were applied in varying combinations and spellings for the systematic search (Table 1):

1. Technology: Here, several terms described above that are found in the literature on CA were used. As the focus of this review is on smart speakers, the search was restricted to this specific type of CA.
2. Product name: As smart speakers were introduced to the market by major American information technology companies, which often use the product names as synonyms for the product, we also included the product or brand names in our search. Globally, Amazon, Google, and Apple are the 3 leading manufacturers; therefore, we included the names of their brands in our search [42].
3. Setting: In order to ensure the most comprehensive search results, we elected to limit our search to the 2 domains of health care and social care without imposing any further restrictions.

**Table 1:** Keywords used in the literature review.

Technology	Vendor, brand, and product	Setting
Smart speaker	Amazon Alexa	Health care
Voice assistant	Amazon Echo	Social care
Voice-based assistant	Apple HomePod	Care
Voice-controlled assistant	Apple Siri	Nursing
Artificial intelligence–driven digital assistant	Google Home	—^a^
Conversational agent	Google Nest	—
Virtual assistant	—	—

^a^Not applicable.

The terms were linked using Boolean operators. Multiple combinations of the search terms were used using different operators (Multimedia Appendix 2).

To select studies relevant to our research interest, we defined the following inclusion criteria for the full-text screening: (1) publications that were released after 2015, as this was the year in which the first commercial smart speaker was introduced to the market, and (2) the use of a smart speaker in health care and social care settings. No restrictions were placed on the specific setting, including hospitals or long-term care facilities. Furthermore, articles were included in which the devices were not implemented in real settings but were developed for specific settings. Studies were excluded if they met one of the following exclusion criteria: (1) papers that do not report on the specific devices that were used (for instance, in some cases, the authors described the use of a personal assistant without explicitly indicating the specific device on which the assistant was operational), (2) studies that did not specifically focus on smart speakers (this encompasses the development of voice-operated applications for use on smartphones or tablets), (3) systematic reviews and other forms of literature-based publications, and (4) articles not published in the English language.

### Process of Study Selection and Data Extraction

We first screened the titles and abstracts for relevance by both authors. No exclusion criteria were applied to the type of publication during the title and abstract search. Should the title or abstract screening indicate the use of a smart speaker in a health care or social care context, the articles were deemed eligible for full-text screening. For the title and abstract screening, as well as the full-text screening, the same 2 authors reviewed each article independently in order to decide on its inclusion or exclusion. In the event of conflicting decisions regarding inclusion or exclusion, the authors attempted to reach a consensus through discussion. As there was no disagreement, there was no need to involve a third party. The data extraction table contains the following information about each article: (1) authors, (2) year of publication, and (3) country of publication. Furthermore, data were collected on the product and the use case. Furthermore, the following aspects were considered: the settings, the target groups, the motivation for using smart speakers, and the limitations of using such a device. As the primary focus was not on methodological aspects, and due to the heterogeneity of the included literature (some described only technical development while others also included user testing and the often-limited reporting of methods), no such information was collected. The articles included were subjected to qualitative thematic analysis in accordance with the methodology outlined in [43]. Using Kuckartz’s [43] approach to qualitative thematic text analysis, researchers identify codes through analysis based on the data gathered. During the process, these codes are then refined. Researchers then identify themes or categories that represent the main findings of the analysis. Identifying themes is a process of examining patterns and similarities between codes and then relating the themes to each other. Consequently, all papers included were read and re-read by both authors, with initial codes being identified. The codes were then compared by the authors, discussed, and grouped into themes. In particular, this included an analysis of the motivation for using the devices and the limitations encountered during the research and development process.

### Ethical Considerations

Given the nature of the study, there were no direct interactions with human participants, and thus, no participants to recruit or consent, and no institutional ethical approval was required.

## Results

### Overview

**Figure 1:**
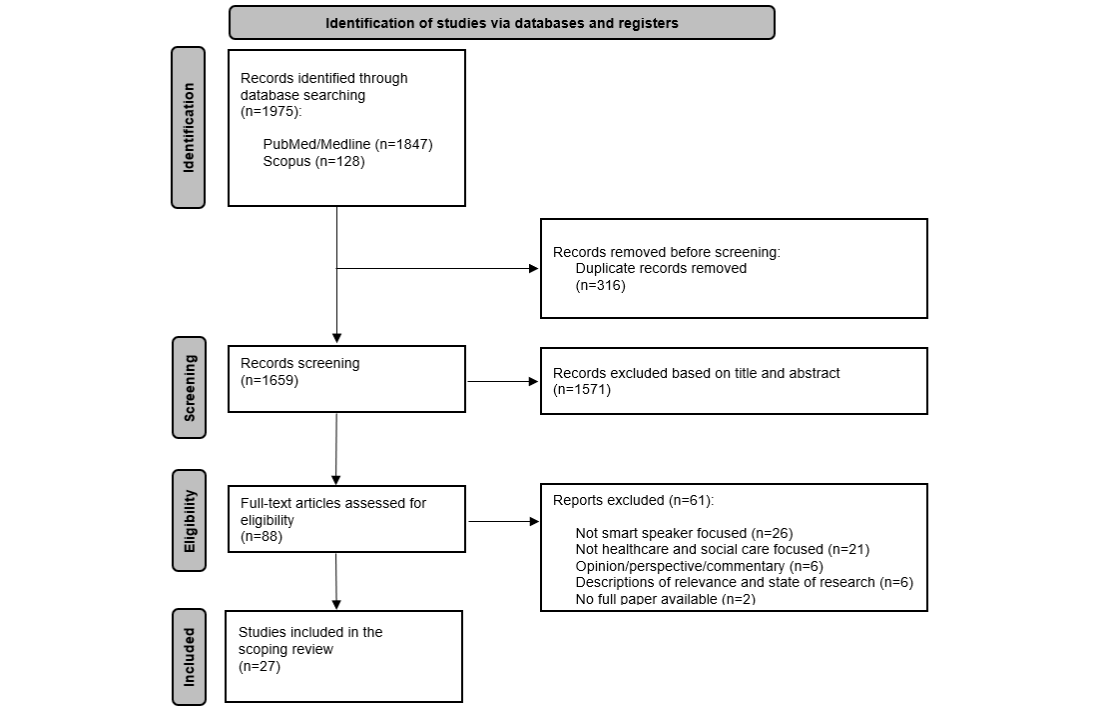
PRISMA (Preferred Reporting Items for Systematic Reviews and Meta-Analyses) flowchart of the search process.

In total, our search yielded 1975 articles. After removing 316 duplicates, 1659 titles and abstracts were screened by the 2 reviewers. The screening of titles and abstracts resulted in the exclusion of 1571 records, leaving 88 full texts to be assessed for eligibility. Of these, 61 articles were excluded, resulting in a final pool of 27 articles for analysis (Figure 1). The data extraction table for the articles included can be found in Multimedia Appendix 3 [3,9,13-15,44-65].

### Year and Country of Publication

The majority of articles included in the analysis were published in the United States (n=15 [9, 15, 39, 45, 46, 49, 51, 53-57, 61, 63, 65]), followed by the United Kingdom (n=4 [3,13,43,62]), North Macedonia (n=2 [58,59]), and Australia (n=2 [52,64]). All articles were published between 2018 and 2022, with 2021 being the year with the highest number of publications, with 11 articles.

### Technology

There was a clear preference for the devices used: Amazon products were used in 23 of the articles, followed by Google (5). A total of 3 papers used a prototype. It should be noted that some articles used devices from several companies. We found 2 types of articles: Those that use the devices, including the infrastructure (eg, frameworks) provided by the developers, and those that mainly use the hardware (eg, for heart rhythm monitoring; Multimedia Appendix 3 [3,9,13-15,44-65]).

The devices were found to be used In 3 main ways: (1) as standard smart speakers without any further modification, for example, to communicate with patients or to support people living alone (for instance, [44,47]); (2) to develop a skill for a specific use case or multiple use cases (for instance, [48]); and (3) to use the smart speaker and, in some cases, the skill to feed information into another system or as a communication device for other systems (for instance, [15]).

### Settings and Target Groups

Given the diverse range of health care and social care settings, we have defined the following categories (Textbox 1). It should be noted that not all articles reported the testing of smart speakers in real health care and social care settings. In some cases, applications were tested in laboratory environments. In the event that this was the case, the intended setting was coded.

**We used the following settings within the domains of health care and special care.**

**Private homes**

- The private living environment includes a person’s own home.

**Hospitals**

- This setting covers acute care hospitals as well as urgent care centers.

**Long-term care facilities**

- This category includes all settings in which long-term care is provided, for example, nursing homes or rehabilitation centers.

**Outpatient services**

- This category covers specialized outpatient services, for example, dental or pain management clinics.

**Other**

- In case the device was tested in a setting not matching the definition of the ones listed above, we categorized it as “other.” For instance, this could be in a car.

Furthermore, 4 target groups were identified. It should be noted that an article can have several target groups, including (1) patients, (2) medical staff members such as physicians, (3) nurses and professional caregivers, and (4) informal caregivers who provide unpaid help to a friend or family member. Moreover, category (5), “other,” was defined for all target groups not matching any of the aforementioned. It should be noted that multiple target groups were covered in one article. Only those who directly interact with the device were included. For instance, Domínguez et al [50] developed a system to support assisted reproduction treatment. Although physicians are involved, only the patients interact with a smart speaker and hence were included.

The most prevalent setting mentioned in the studies included was home care (n=20), followed by hospitals (n=6). Outpatient care (n=3) was less frequently observed (Multimedia Appendix 3 [3,9,13-15,44-65]). In one instance, the setting was not specified [14]. However, it is best classified under home care.

Among the target groups, patients are the most frequent users mentioned in 23 of the articles (Multimedia Appendix 3 [3,9,13-15,44-65]). Older adults, in particular, were often seen as a promising target group, and we found that 11 of the included publications focus on this target group [66] (Multimedia Appendix 3 [3,9,13-15,44-65]). While some articles included descriptions of the development and testing of skills specifically designed for older adults [51,52], others explored the general acceptance and potential of the technology for older adults. For instance, Lee et al [51] developed multiple skills aimed at older persons, including a reminder to take medication, a diet tracking system, and a skill alerting caregivers in case of a fall. Nallam et al [49] simulated a CA to answer health-related questions asked by older persons. O’Brien et al [47] used off-the-shelf devices without any form of modification to investigate the effects on home-bound older adults with social isolation. The participants used the devices for a variety of purposes, including monitoring their health and well-being, as well as for emergency communication. Some authors report that older adults constitute the largest group of first adopters of smart speakers. In addition, smart speakers allow easy contact with caregivers [12] or low-threshold access to health information [13]. Older adults as potential users of CA have been the focus before [39,67,68].

The second most frequent target group was physicians (n=11), followed by other health professionals (eg, nurses; n=9) and informal caregivers (n=1; Multimedia Appendix 3 [3,9,13-15,44-65]). These results demonstrate that the majority of articles focus on supporting nonresidential care.

Table 2 provides an overview of all settings and target groups. It is important to note that a single paper can include multiple settings and target groups.

While previous statements covered the number of papers included in the review, Table 2 combines user groups and settings across the studies covered. It shows that patients are the most common target group, while home care is the most common setting.

**Table 2:** Settings and user groups.

	Patients	Physicians	Older adults	Nurses and so on	Informal caregivers	Other	Total
Home care	19	5	11	7	1	1	44
Hospitals	4	5	0	2	0	0	11
Outpatient care	2	2	1	1	0	0	6
Total	25	12	12	10	1	1	

### Use Cases

We found several use cases covering, among others, hearing tests [14], cardiovascular diseases [15,46], pregnancy companion [13], cancer management [eg, 58,59], or medication reminders [69]. It must be noted that several articles reported that smart speakers were used in multiple use cases. For example, Wright [70] describes that a local authority was involved in developing applications, including “a Skill that prompted users to take their medicine; a Skill that helped to record and manage care tasks; a Skill to facilitate communication with caregivers by recording messages; and a Skill to connect users to a trusted LA directory of services” [44]. Jadczyk et al [71], who developed a voice-enabled automated platform for the collection of medical data from patients with cardiovascular disease, describe 5 use cases within their study: (1) education, (2) process optimization, (3) patient support, and (4) data collection, and (5) medical device grade solutions (eg, diagnose and treatment). The devices were used to open patient files and images, initiate conference calls, or record images and videos [4].

While most of the identified use cases were found in the domain of health care, social care played a subordinate role. Still, we found several articles reporting on the use of smart speakers in this domain. Within this field, elderly care was the most relevant area. For instance, O’Brien et al [47] use a smart speaker to reduce loneliness and social isolation among older adults living at home. Palumbo et al [72] developed personalized coaching for older individuals to increase their well-being by aiming at the areas of physical activity, nutrition, cognition, and social relationships. In the domain of social care, older adults living at home or care home residents were the main user group (eg, [3]).

### Motivation for Use

The reasons for using smart speakers in health care are framed with various arguments. Besides their low acquisition costs [51], this also includes aspects applying to digital technologies in health care and social care in general, such as the possibility to deliver care remotely without restrictions in time and space (eg, Sadavarte et al [13]). Another motivation is the fact that smart speakers are already widely accepted as a consumer technology [45,52]. Hence, users already know how to operate the devices and are also familiar with their limitations. Other aspects cover potentially increased productivity across the use cases that we identified. For instance, Bhatt et al [45] used a voice-based assistant to access and update an electronic health record. They see advantages in terms of efficiency (less time spent on data input) and accuracy, as speech-to-text might result in fewer errors. Ultimately, this might also benefit patients as waiting time is reduced [45]. Jadczyk et al [71] highlighted the main potential in the possibility of automating traditional telehealth services: “Voice chatbots can support routine care through automatic at-home monitoring, triaging, screening, providing medical recommendations and guidelines, and improving operational workflow” [15,71].

Another advantage is the user interface, which is easy to navigate [11]. Cheng et al [55] argue that the main advantage of the technology is that it: “eliminate[s] the struggles that are associated with strictly tactile screens.” (2018); or that human-like verbal communication that feels more natural and intuitive and particularly that the devices can be used hands-free [55]. Jansons et al [52] drive on the research of Foehr and Germelmann [73] and argue that the devices “may enhance adherence to remotely-delivered exercise interventions […], because the human-like attributes associated with these technologies may elicit a sense of familiarity, social presence, and human engagement” [52]. Moreover, the authors see this as an advantage for older users [53] who support this viewpoint and argue that “digital non-natives” might be especially benefitting from this technology. For instance, Kim [4] tested the experiences of older adults who used the devices for the first time and found that due to the simple interaction, health-related questions were a typical use case.

The form of smart speakers and their design were mentioned in some publications. Gouda et al [74] saw the fact that smart speakers are “non-invasive” technology as a main advantage. As the devices can be placed nearly anywhere in the room and can be operated without the need to see them, it allows for new ways of interaction. Luo et al [56] also see a benefit in the fact that the immobility of the devices is as helpful as this helps, in contrast to mobile phones, in establishing habits and routines.

Wright [44] describes the use of smart speakers in trials run by local authorities in England. Drawing on interviews with managers from 8 English local authorities, benefits are seen in the low-cost supplement or alternative to telecare. Or, as one of his interview partners put it: “have the advantages of being sophisticated and powerful, relatively cheap, already widely used and familiar, designed with a degree of accessibility and intuitive use in mind, and a growing level of interoperability with other networked digital devices aided by an open development framework” [44]. One of the results of the study is that local authorities chose Amazon’s Echo because of “councils facing depleted funds, a lack of expert guidance on care technologies, and an increasingly complex and fragmented care technology marketplace” [44].

### Limitations of Smart Speakers

In addition, various limitations of the technology were addressed in the included articles. Here, most technical limitations were named (1) insufficient hearing comprehension [57], speech recognition [51], or emotion recognition [54]; (2) that there is no interruption of the recording during slow speeches allowed [14]; (3) difficult functioning in the natural living environments due to interfering noises [3]; (4) that the correctness of the answer is not always accurate [51]; and (5) that the devices allow longer conversations [49]. Internet access must also be provided [48,75]. Besides these technical aspects, there were also social aspects mentioned. This covered the (lack of) user acceptance, particularly among older users and professional caregivers [45,76], but also their lack of basic digital skills [75]. These supposedly low digital skills might lead to challenges in interacting with the devices. Users might forget the wake word, there may be timing issues when communicating with the devices, or they might have difficulties in setting up the devices [47,53]. Another issue that was mentioned regularly was data protection. Here, the misuse of sensitive data is particularly pointed out. For example, if security measures are inadequate, it would be possible to manipulate the medication and thus actively harm the patient [12]. Cheng et al [55] also argue for multimodal solutions as people might feel uncomfortable talking to devices in front of other people.

## Discussion

### Principal Findings

Our aim was to identify use cases and scenarios in which smart speakers can be used within health care and social care. The results show that smart speakers are used in various contexts and for multiple reasons. The main features used are NLP and hands-free interaction. Moreover, the fact that the technology is widely used in private homes and hence many persons are used to interact with the devices are important aspects. In addition to offering relatively inexpensive hardware, smart speakers and the companies behind them provide software frameworks and infrastructure, such as Amazon’s skill, which assists developers in the design and marketing of their products.

It is important to note that there is no clear definition of smart speakers. One challenge of this study was the varying definitions of the technology, with the term often being used interchangeably with personal assistants such as Siri or Cortana. These assistants play an important role in the use of smart speakers, which arguably only serve as a shell equipped with microphones and loudspeakers for them. However, we argue that smart speakers should be considered a distinct technology. Based on this review, we understand smart speakers as a type of CA bound to a fixed location. Within the field of health care and social care, the technology can be used in various settings and use cases such as communication, documentation, or diagnosis and therapy of diseases hands-free. Smart speakers are equipped with microphones and loudspeakers and connected to the internet. They usually come with an integrated digital assistant, but even without such an assistant, they offer multiple features that can be used across various settings. Smart speakers can be customized using either skills or apps that can be installed on the devices.

The results show that all publications were published between 2018 and 2021. Furthermore, the majority were published in the United States. The following explanations can be given for these 2 results. Alexa was the first voice assistant that was compliant with the Health Insurance Portability and Accountability Act (HIPAA), allowing it to be the access example of clinical records. In England, the National Health Service contracted with Amazon to enable Alexa in 2019 to answer health-related questions, raising questions about privacy and how health care data would be used [44,45]. The HIPAA compliance and the fact that the National Health Service contracted with Amazon explains why most studies have been carried out in the United States and the United Kingdom. Arguably, European countries are not as present due to more strict data protection regulations. Moreover, the use of smart speakers is significantly higher in the United States than in other countries, which in turn could also be related to data protection regulations [77]. Interestingly, Asian countries have, with few exceptions, also not been represented in the included articles. This seems counterintuitive as, in terms of market sales, smart speaker technology by Asian technology companies is more and more successful [42].

It also became clear that the devices were clearly dominant in the publications. This should be criticized from a scientific point of view. We were able to identify the following explanations for this result.

Since Amazon entered the market in 2015 and continuously updates its product line, off-the-shelf devices have recently increased in terms of market penetration, making them more popular for research and development. That Amazon’s Echo was used in the vast majority of articles included comes as no surprise, and Amazon’s market dominance is based on several factors. First, the company was the first to release a smart speaker to consumers. Second, Amazon’s voice assistant, Alexa, has been embedded in a broad range of devices, including wall clocks, by third-party manufacturers. Third, Amazon sells products of the Echo family at comparably low prices, starting at around US $20. Fourth, Amazon offers an infrastructure through its Skill Store and several frameworks for developers. Fifth, in the United States, the Echo is HIPAA-compliant.

The dominance of Amazon’s smart speaker in the included papers poses several risks depending on the use case, some of which are discussed in the papers themselves. In terms of the devices themselves in their off-the-shelf version, the interaction is limited. For example, Nallam et al [49] used a smart speaker prototype as they argue that developed solutions often do not support conversational interactions and explore scenarios that are not yet supported.

The articles included in this publication address a diverse range of use cases across various settings, thereby demonstrating the versatility of smart speakers and the technology of NLP and AI incorporated in them. This technology can be used in a multitude of contexts within the domains of health care and social care. Overall, 2 general use cases can be distinguished: (1) supporting patients and their relatives in their private living environments and (2) supporting professional health care workers in clinical settings. As the devices were originally developed for private home environments and primarily for entertainment and e-commerce applications, it is unsurprising that this setting was the dominant one across the papers included in this review. This could be seen as an indicator of the restructuring of health care services, with an increased focus on the private living environment. Several clinical use cases supported by smart speakers could be automated and not be restricted to clinical settings (eg, [14,48]). Only in a few cases does the paper focus on clinical use cases and professional personnel (eg, [4,45,71]).

That patients, and particularly older adults, were the main target group supports this conclusion. Moreover, this also underlines that the role of patients and practices of health and care change against the background of digitalization and the use of AI [78]. While some of the use cases identified were exclusively designed for clinical settings, the majority can, in theory, be implemented in multiple settings. This could support patient empowerment, as smart speakers can be used to support the household as a central place of health care. An argument supporting the fit of the devices for older adults is that smart speakers do not require “reasonable levels of vision and manual dexterity” [79,80].

A key rationale for using the devices is not only their competitive pricing but also the potential to reduce expenditure by enhancing the efficiency of staff members and care processes, for instance, through enhanced documentation or facilitating straightforward communication with patients, colleagues, or clients. Although the majority of the papers reviewed argue that smart speakers could provide such benefits, these potential benefits depend on several circumstances. The first is whether the devices can be installed as they are or whether new skills or, more complexly, additional hardware or modifications are required. This depends on the use case and also the target group. Although many people are used to interacting with the devices, older adults might not have any experience and could need training.

The majority of the papers in our sample can be classified as exploratory in nature. The research designs used are predominantly qualitative, with sample sizes that are relatively small and no long-term studies conducted in real-world scenarios. This underscores the fact that the technology itself is still relatively new, particularly within the context of health care and social care. In addition, researchers and developers are still exploring the technology’s potential applications in health care and social care, which may have become more apparent in the context of the pandemic. Both sectors are currently experiencing financial strain due to rising expenditure and a shortage of qualified personnel [81]. New technologies are frequently viewed as a potential solution to these challenges [70].

Smart speakers and digital voice assistants like Alexa are quite limited in terms of their initial dialogue management, which can be seen as an important motivator to using the systems as they are easier to develop and control. This finding is in line with a systematic review of CA in health care carried out by Laranjo et al [1]. The authors could identify 17 articles using 14 different CA. Most papers covered by the review evaluated task-oriented CA that aims at supporting patients and clinicians. Systems allowing the management of complex dialogues were only identified in 1 case. Even though conversational systems have proven to be beneficial for health-related purposes, most assistants allow only constrained user input (eg, multiple-choice answers) [1,82]. Clark et al [83] argue that users interact in “clearly delineated task-based conversations” and “fall short of reflexive and adaptive interactivity.” According to the authors, the term conversation is “a poor description of the current interaction experience” with an AI using common smart speakers [83]. Hence, they suggest testing “human-agent interaction as a new genre of conversation, with its own rules, norms and expectations” [83]. The devices have only a limited capability to actually be able to engage in a conversational dialogue. Conversations are task-oriented instead of offering interactions initiated by the user and not by the device. While this might be true, it seems to be only a matter of time before future updates might be used to allow more natural dialogues, as is already the case with generative AI such as ChatGPT.

The analysis showed that change in existing practices and routines is an important aspect. Drawing on Sezgin et al [84], Capasso and Umbrello [85] argue that the novelty of CAs is that they act as “intermediaries between the health care system as a whole and the public,” changing practices in health care and social care. Here, several studies follow the normative aim to implement innovative technologies in order to improve processes and outcomes. The use of smart speakers—or CAs in general—follows a technology-driven approach. Already existing technologies are transferred to the domains of health and social care. Due to the exploratory design of most studies, the emphasis is put on the technology and not on the context, like organizational or social factors. The logic of a “fitting” technology seems to be a main driver of many studies, neglecting the analysis of potentially changing social practices.

The dominance of Amazon in our sample has to be seen from a critical perspective. The company itself began offering the service Alexa Together and was able to emulate existing approaches and leverage its financial and market clout to challenge competitors. Moreover, developers depend on the technology, that is, the hardware and also the software frameworks of one company. As a consequence, the dominant position of Amazon might increase due to research using the company’s products. If only one product from a particular company is examined, the capabilities of other products are not taken into account, as they may perform better, for example, and might be used to copy promising applications.

### Limitations

This paper has several limitations. First, the number of databases searched. To address this limitation, a cross-search was performed in Google Scholar to rule out the possibility that important articles were not found. In addition, to broaden the search strategy, other forms of literature, such as trial reports, could be included in future studies. For instance, a few trials using smart speakers are registered on clinicaltrials.gov. However, we decided not to include these as they did not provide all the information we wanted to obtain (eg, motivations for using the devices). Second, we restricted our search to the English language only. Few papers were found from the Asian region, probably due to the language limitation of the search. This limitation was mitigated by using brand names as search terms focusing on the brands with the highest market share. However, as recent market research shows, there is a shift toward products developed in Asian countries, and future studies should include a wider range of brands and products. Another limitation is that we only looked at smart speakers, which excludes other voice assistants that use essentially the same technology (such as digital assistants on smartphones and tablets). We deliberately excluded these as this review focused specifically on smart speakers as a form of CA, and we argue that the technology of smart speakers needs to be seen as a technology in its own right.

### Conclusion

In this paper, a scoping review was conducted on the use of smart speakers in health care and social care settings. The analysis showed that—due to the widespread use of devices like Amazon’s Echo—smart speaker technology has been tested and implemented in various settings and use cases in the health and social care sectors. The main setting was the private home environment, and the main user group was patients. There are, however, also approaches to making use of the technology in other settings, such as hospitals. It seems likely that due to technical progress in the field of AI and the market power of the companies behind the devices, there will be more use cases of smart speakers in the (near) future.

## Appendix

Multimedia Appendix 1 Preferred Reporting Items for Systematic reviews and Meta-Analyses extension for Scoping Reviews (PRISMA-ScR) checklist.

Multimedia Appendix 2 Database search details.

Multimedia Appendix 3 Data extraction table.

## Abbreviations

AI: artificial intelligence
CA: conversational agent
HIPAA: Health Insurance Portability and Accountability Act
NLP: natural language processing
PRISMA: Preferred Reporting Items for Systematic Reviews and Meta-Analyses
PRISMA-ScR: Preferred Reporting Items for Systematic Reviews and Meta-Analyses Extension for Scoping Reviews
SR: speech recognition

## Data Availability

The datasets generated during and/or analyzed during this study are available from the corresponding author upon reasonable request.
