# Supplementary material for: Identification of Use Cases, Target Groups, and Motivations Around Adopting Smart Speakers for Health Care and Social Care Settings: Scoping Review
Source: JMIR AI. 2025 Jan 13;4:e55673. doi: 10.2196/55673 (PMC11773277; doi:10.2196/55673)
Supplement: Multimedia Appendix 2 [file ai_v4i1e55673_app2.docx]

**Appendix 2:** Database search details

| **PubMed/ Medline** |  |
| --- | --- |
| **Search terms** | **Number of articles** |
| ***PubMed*** |  |
| (("smart speaker"[Title/Abstract]) AND ("healthcare" [Title/Abstract])) | 3 |
| “smart speaker” and “healthcare” | 6 |
| "voice assistant” AND "healthcare" | 17 |
| (("smart speaker"[Title/Abstract]) AND ("social care" [Title/Abstract])) | 4 |
| (("Virtual assistant"[Title/Abstract]) AND ("social care" [Title/Abstract])) | 1 |
| (("Virtual assistant"[Title/Abstract]) AND ("healthcare" [Title/Abstract])) | 17 |
| (("Conversational agent"[Title/Abstract]) AND ("healthcare" [Title/Abstract])) | 30 |
| (("amazon alexa"[Title/Abstract]) AND ("healthcare" [Title/Abstract])) | 9 |
| (("amazon alexa"[Title/Abstract]) AND ("care" [Title/Abstract])) | 8 |
| (("apple homepod"[Title/Abstract]) AND ("healthcare" [Title/Abstract])) | 347 |
| (("apple homepod"[Title/Abstract]) AND ("care" [Title/Abstract])) | 6 |
| (("apple homepod"[Title/Abstract]) AND ("nursing" [Title/Abstract])) | 169 |
| (("google home"[Title/Abstract]) AND ("care" [Title/Abstract])) | 4 |
| (("siri"[Title/Abstract]) AND ("healthcare" [Title/Abstract])) | 21 |
| "siri" AND "healthcare" | 68 |
| "siri" AND "care" | 128 |
| (("siri"[Title/Abstract]) AND ("care" [Title/Abstract])) | 54 |
| "siri" and "nursing" | 47 |
| ***Medline*** |  |
| (("amazon alexa"[Title/Abstract]) AND ("care" [Title/Abstract])) | 8 |
| "amazon alexa" and "care" | 9 |
| (("amazon alexa"[Title/Abstract]) AND ("healthcare" [Title/Abstract])) | 5 |
| (("amazon echo"[Title/Abstract]) AND ("care" [Title/Abstract])) | 4 |
| (("google home"[Title/Abstract]) AND ("care" [Title/Abstract])) | 4 |
| (("google home"[Title/Abstract]) AND ("nursing" [Title/Abstract])) | 1 |
| (("voice assistant"[Title/Abstract]) AND ("nursing" [Title/Abstract])) | 1 |
| "voice assistent" and "nursing" | 2 |
| (("amazon echo"[Title/Abstract]) AND ("care" [Title/Abstract])) | 4 |
| voice-based assistant | 26 |
| voice-based assistant healthcare | 2 |
| voice-controlled assistant | 55 |
| voice-controlled assistant Social care | 5 |
| voice-controlled assistant healthcare | 2 |
| AI-driven digital assistant | 10 |
| AI-driven digital assistant Social care | 1 |
| Conversational agent healthcare | 379 |
| Conversational agent Social care | 186 |
| Virtual assistant healthcare Social care | 42 |
|  | |
| **Scopus with Article title, Abstract, Keywords** | |
| **Search terms** | **Number of articles** |
| "alexa" AND "healthcare" | 66 |
| "amazon alexa" AND "nursing" | 3 |
| "amazon alexa" AND "healthcare" | 26 |
| "google home" AND "nursing" | 1 |
| "smart speaker" AND "nursing" | 6 |
| "voice assistant" AND "nursing" | 6 |
| "siri" AND "nursing" | 64 |
| "Apple siri" AND "care" | 17 |
| **Sum** | **1873** |
