# Supplementary material for: Identification of Use Cases, Target Groups, and Motivations Around Adopting Smart Speakers for Health Care and Social Care Settings: Scoping Review
Source: JMIR AI. 2025 Jan 13;4:e55673. doi: 10.2196/55673 (PMC11773277; doi:10.2196/55673)
Supplement: Multimedia Appendix 3 [file ai_v4i1e55673_app3.docx]

**Table S1.**

| **#** | **Author** | **Title** | **Year** | **Country** | **Product** | **Settings** | **Target groups** | **Older adults** |
| --- | --- | --- | --- | --- | --- | --- | --- | --- |
| 1 | Arem et al [57] | Assessing Breast Cancer Survivors’ Perceptions of Using Voice-Activated Technology to Address Insomnia: Feasibility Study Featuring Focus Groups and In-Depth Interviews | 2020 | USA | Amazon Echo | Home care | Patients |  |
| 2 | Bhatt et al [45] | DocPal: A voice-based EHR assistant for health practitioners | 2021 | USA | Amazon Echo | Hospital | Physicians |  |
| 3 | Lee et al [51] | Eldercare Robotics - Alexa | 2020 | USA | Amazon Echo | Home care | Patients | X |
| 4 | Jadczyk et al [15] | Feasibility of a voice-enabled automated platform for medical data collection: CardioCube | 2019 | USA | Amazon Echo | Hospital | Patients, Physicians, Nurses etc. |  |
| 5 | Dojchinovski et al [58] | Interactive home healthcare system with integrated voice assistant | 2019 | Nordmazedonien | Amazon Echo, Google Home | Home care | Patients | X |
| 6 | Ilievski et al [79] | Interactive voice assisted home healthcare systems | 2019 | Nordmazedonien | Amazon Echo, Google Home | Home care | Patients, Physicians, Nurses etc. | X |
| 7 | Domínguez et al [59] | IoMT-Driven eHealth: A Technological Innovation Proposal Based on Smart Speakers | 2020 | Spain | Amazon Echo | Home care | Patients, Physicians, Nurses etc. |  |
| 8 | Thomas [54] | Patient and clinician-centric healthcare enhancement through speech recognition: A research proposal | 2019 | USA | Amazon Echo | Outpatient care, Hospital | Patients, Physicians, Nurses etc. |  |
| 9 | Sadavarte and Bodanese [13] | Pregnancy Companion Chatbot Using Alexa and Amazon Web Services | 2019 | UK | Amazon Echo | Home care | Patients |  |
| 10 | Wright [44] | The Alexafication of Adult Social Care: Virtual Assistants and the Changing Role of Local Government in England | 2021 | UK | Amazon Echo | Home care | Patients, Physicians, Nurses etc. | X |
| 11 | Wang et al [46] | Using smart speakers to contactlessly monitor heart rhythms | 2021 | USA | Amazon Echo, Google Home | Home care | Patients |  |
| 12 | Nallam et al [49] | A Question of Access: Exploring the Perceived Benefits and Barriers of Intelligent Voice Assistants for Improving Access to Consumer Health Resources Among Low-Income Older Adults | 2020 | USA | Prototype | Home care | Patients | X |
| 13 | Yoo et al [60] | Deep learning-based smart speaker to confirm surgical sites for cataract surgeries: A pilot study. | 2020 | South Korea | Prototype | Hospital, Outpatient care | Physicians |  |
| 14 | Ismail et al [61] | Feasibility of Use of a Smart Speaker to Administer Snellen Visual Acuity Examinations in a Clinical Setting. | 2020 | USA | Amazon Echo | Hospital | Patients |  |
| 15 | Corbett et al [9] | Medication Adherence Reminder System for Virtual Home Assistants: Mixed Methods Evaluation Study. | 2021 | USA | Amazon Echo | Home care | Patients |  |
| 16 | Qiu et al [53] | Nurse AMIE: Using Smart Speakers to Provide Supportive Care Intervention for Women with Metastatic Breast Cancer | 2021 | USA | Amazon Echo | Home care, Outpatient care | Patients | X |
| 17 | Chambers, R.; Beaney, P. [62] | The potential of placing a digital assistant in patients’ homes | 2019 | UK | Amazon Echo | Home care | Patients |  |
| 18 | Ooster et al [14] | “Computer, test my hearing”: Accurate speech audiometry with smart speakers | 2019 | Germany | Amazon Echo | Home care | Patients, Physicians |  |
| 19 | Sharma et al [48] | Voice-based screening for SARS-CoV-2 exposure in cardiovascular clinics | 2021 | Canada | Amazon Echo | Hospital | Patients |  |
| 20 | Kim et al [63] | Development of a Smart Hospital Assistant: Integrating Artificial Intelligence and a Voice-User Interface for Improved Surgical Outcomes | 2021 | USA | Amazon Echo | Hospital | Physicians |  |
| 21 | Jansons et al [52] | Barriers and enablers for older adults participating in a home-based pragmatic exercise program delivered and monitored by Amazon Alexa: a qualitative study | 2022 | Australia | Amazon Echo | Home care | Physicians, Nurses etc. | X |
| 22 | Jansons et al [64] | Delivery of Home-Based Exercise Interventions in Older Adults Facilitated by Amazon Alexa: A 12-week Feasibility Trial | 2021 | Australia | Amazon Echo | Home care | Patients, Nurses etc. | X |
| 23 | O'Brien et al [47] | Optimizing voice-controlled intelligent personal assistants for use by home-bound older adults | 2022 | USA | Google Home | Home care | Patients, Physicians, Nurses etc., Other | X |
| 24 | Apergi et al [65] | Voice Interface Technology Adoption by Patients With Heart Failure: Pilot Comparison Study | 2021 | USA | Amazon Echo | Home care | Patients |  |
| 25 | Cheng et al [55] | Development and evaluation of a healthy coping voice interface application using the Google home for elderly patients with type 2 diabetes. | 2018 | USA | Google Home | Home care | Patients | X |
| 26 | Edwards et al [3] | The Use of Smart Speakers in Care Home Residents: Implementation Study | 2021 | UK | Amazon Echo | Home care | Patients, Nurses etc., Informal caregivers | X |
| 27 | Luo et al [56] | Tandem Track: shaping consistent exercise experience by complementing a mobile app with a smart speaker. | 2020 | USA | Amazon Echo | Home care | Patients |  |

### **References**

3. Edwards KJ, Jones RB, Shenton D, et al. The Use of Smart Speakers in Care Home Residents: Implementation Study. Journal of medical Internet research 2021;**23**(12):e26767. doi:[10.2196/26767](https://www.jmir.org/2021/12/e26767) [published Online First: 20 December 2021].

9. Corbett CF, Combs EM, Chandarana PS, et al. Medication Adherence Reminder System for Virtual Home Assistants: Mixed Methods Evaluation Study. JMIR formative research 2021;**5**(7):e27327. doi:[10.2196/27327](https://formative.jmir.org/2021/7/e27327) [published Online First: 13 July 2021].

13. Sadavarte SS, Bodanese E. Pregnancy Companion Chatbot Using Alexa and Amazon Web Services. In: 2019 IEEE Pune Section International Conference (PuneCon): IEEE 2019:1-5. doi: [10.1109/punecon46936.2019.9105762](https://ieeexplore.ieee.org/document/9105762)

14. Ooster J, Moreta PNP, Bach J-H, et al. “Computer, Test My Hearing”: Accurate Speech Audiometry with Smart Speakers. In: Interspeech 2019. ISCA: ISCA 2019:4095-4099. doi: [10.21437/interspeech.2019-2118](https://www.isca-archive.org/interspeech_2019/ooster19_interspeech.html)

15. Jadczyk T, Kiwic O, Khandwalla RM, et al. Feasibility of a voice-enabled automated platform for medical data collection: CardioCube. International journal of medical informatics 2019;**129**:388-393. doi:[10.1016/j.ijmedinf.2019.07.001](https://www.sciencedirect.com/science/article/abs/pii/S1386505619303417?via%3Dihub) [published Online First: 4 July 2019].

44. Wright J. The Alexafication of Adult Social Care: Virtual Assistants and the Changing Role of Local Government in England. International journal of environmental research and public health 2021;**18**(2). doi:[10.3390/ijerph18020812](https://www.mdpi.com/1660-4601/18/2/812) [published Online First: 19 January 2021].

45. Bhatt V, Li J, Maharjan B. DocPal: A Voice-based EHR Assistant for Health Practitioners. In: 2020 IEEE International Conference on E-health Networking, Application & Services (HEALTHCOM): IEEE 2021:1-6. doi: [10.1109/healthcom49281.2021.9399013](https://ieeexplore.ieee.org/document/9399013)

46. Wang A, Nguyen D, Sridhar AR, et al. Using smart speakers to contactlessly monitor heart rhythms. Communications biology 2021;**4**(1):319. doi:[10.1038/s42003-021-01824-9](https://www.nature.com/articles/s42003-021-01824-9) [published Online First: 9 March 2021].

47. O'Brien K, Light SW, Bradley S, et al. Optimizing voice-controlled intelligent personal assistants for use by home-bound older adults. Journal of the American Geriatrics Society 2022;**70**(5):1504-1509. doi:[10.1111/jgs.17625](https://agsjournals.onlinelibrary.wiley.com/doi/10.1111/jgs.17625) [published Online First: 14 January 2022].

48. Sharma A, Oulousian E, Ni J, et al. Voice-based screening for SARS-CoV-2 exposure in cardiovascular clinics. European heart journal. Digital health 2021;**2**(3):521-527. doi:[10.1093/ehjdh/ztab055](https://academic.oup.com/ehjdh/article/2/3/521/6300520) [published Online First: 16 June 2021].

49. Nallam P, Bhandari S, Sanders J, et al. A Question of Access: Exploring the Perceived Benefits and Barriers of Intelligent Voice Assistants for Improving Access to Consumer Health Resources Among Low-Income Older Adults. Gerontology and Geriatric Medicine 2020;**6**:2333721420985975. doi:[10.1177/2333721420985975](https://journals.sagepub.com/doi/10.1177/2333721420985975) [published Online First: 29 December 2020].

50. Domínguez D, Morales L, Sánchez N, et al. IoMT-Driven eHealth: A Technological Innovation Proposal Based on Smart Speakers. In: Rojas I, Valenzuela O, Rojas F, et al., eds. Bioinformatics and Biomedical Engineering. Cham: Springer International Publishing 2020:378-386.

51. Lee E, Vesonder G, Wendel E. Eldercare Robotics - Alexa. In: 2020 11th IEEE Annual Ubiquitous Computing, Electronics & Mobile Communication Conference (UEMCON): IEEE 2020:820-825. doi: [10.1109/uemcon51285.2020.9298147](https://ieeexplore.ieee.org/document/9298147)

52. Jansons P, Fyfe J, Via JD, et al. Barriers and enablers for older adults participating in a home-based pragmatic exercise program delivered and monitored by Amazon Alexa: a qualitative study. BMC geriatrics 2022;**22**(1):248. doi:[10.1186/s12877-022-02963-2](https://bmcgeriatr.biomedcentral.com/articles/10.1186/s12877-022-02963-2) [published Online First: 25 March 2022].

53. Qiu L, Kanski B, Doerksen S, et al. Nurse AMIE: Using Smart Speakers to Provide Supportive Care Intervention for Women with Metastatic Breast Cancer. In: Kitamura Y, Quigley A, Isbister K, et al., eds. Extended Abstracts of the 2021 CHI Conference on Human Factors in Computing Systems. New York, NY, USA: ACM 2021:1-7. doi: [10.1145/3411763.3451827](https://dl.acm.org/doi/10.1145/3411763.3451827)

54. Thomas G. Patient and Clinician-Centric Healthcare Enhancement through Speech Recognition: A Research Proposal 2019. doi: 10.5176/2301-394X_ACE19.581

55. Cheng A, Raghavaraju V, Kanugo J, et al. Development and evaluation of a healthy coping voice interface application using the Google home for elderly patients with type 2 diabetes. In: 2018 15th IEEE Annual Consumer Communications & Networking Conference (CCNC): IEEE 2018:1-5. doi: [10.1109/ccnc.2018.8319283](https://ieeexplore.ieee.org/document/8319283)

56. Luo Y, Lee B, Choe EK. TandemTrack: Shaping Consistent Exercise Experience by Complementing a Mobile App with a Smart Speaker. 2020. Presented at: CHI '20: Proceedings of the 2020 CHI Conference on Human Factors in Computing Systems; April 23, 2020; Honolulu, HI. doi: [10.1145/3313831.3376616](https://dl.acm.org/doi/10.1145/3313831.3376616) [accessed 2024-12-22 ]

57. Arem H, Scott R, Greenberg D, et al. Assessing Breast Cancer Survivors' Perceptions of Using Voice-Activated Technology to Address Insomnia: Feasibility Study Featuring Focus Groups and In-Depth Interviews. JMIR cancer 2020;**6**(1):e15859. doi:[10.2196/15859](https://cancer.jmir.org/2020/1/e15859/) [published Online First: 26 May 2020].

58. Dojchinovski D, Ilievski A, Gusev M. Interactive home healthcare system with integrated voice assistant. 2019. Presented at: 42nd International Convention on Information and Communication Technology, Electronics and Microelectronics (MIPRO); May 20-24, 2019; Opatija, Croatia. doi: [10.23919/MIPRO.2019.8756983](https://ieeexplore.ieee.org/document/8756983) [accessed 2024-12-22 ]

59. Ilievski A, Dojchinovski D, Gusev M. Interactive voice assisted home healthcare systems. 2019. Presented at: BCI'19: 9th Balkan Conference in Informatics; September 26-28, 2019; Sofia, Bulgaria. doi: [10.1145/3351556.3351572](https://dl.acm.org/doi/10.1145/3351556.3351572) [accessed 2024-12-22 ]

60. Yoo TK, Oh E, Kim hk, Ryu IH, Lee IS, Kim JS, Kim JK. Deep learning-based smart speaker to confirm surgical sites for cataract surgeries: a pilot study. PLoS One 2020; 15(4):e0231322. doi: [10.1371/journal.pone.0231322](https://journals.plos.org/plosone/article?id=10.1371/journal.pone.0231322)

61. Ismail HO, Moses AR, Tadrus M, Mohamed EA, Jones LS. Feasibility of use of a smart speaker to administer Snellen visual acuity examinations in a clinical setting. JAMA Netw Open 2020 Aug 03; 3(8):e2013908. doi: [10.1001/jamanetworkopen.2020.13908](https://jamanetwork.com/journals/jamanetworkopen/fullarticle/2769502)

62. Chambers R, Beaney P. The potential of placing a digital assistant in patients' homes. Br J Gen Pract 2020 Jan; 70(690):8-9. doi: [10.3399/bjgp20X707273](https://bjgp.org/content/70/690/8)

63. Kim JH, Um R, Liu J, Patel J, Curry E, Aghabaglou F, Mahapatra S, Ainechi A, Tsehay Y, Ehresman J, Hwang B, Tyler B, Iyer R, Theodore N, Manbachi A. Development of a smart hospital assistant: integrating artificial intelligence and a voice-user interface for improved surgical outcomes. Proc SPIE Int Soc Opt Eng 2021 Feb; 11601. doi: [10.1117/12.2580995](https://www.spiedigitallibrary.org/conference-proceedings-of-spie/11601/2580995/The-development-of-Smart-Hospital-Assistant--integrating-artificial-intelligence/10.1117/12.2580995.short)

64. Jansons P, Dalla Via J, Daly RM, Fyfe JJ, Gvozdenko E, Scott D. Delivery of home-based exercise interventions in older adults facilitated by Amazon Alexa: a 12-week feasibility trial. J Nutr Health Aging 2022; 26(1):96-102. doi: [10.1007/s12603-021-1717-0](https://www.sciencedirect.com/science/article/pii/S1279770723009089?via%3Dihub)

65. Apergi LA, Bjarnadottir MV, Baras JS, Golden BL, Anderson KM, Chou J, Shara N. Voice interface technology adoption by patients with heart failure: pilot comparison study. JMIR Mhealth Uhealth 2021 Apr 01; 9(4):e24646. doi: [10.2196/24646](https://mhealth.jmir.org/2021/4/e24646)
